# Ethical aspects of human biobanks: a systematic review

**DOI:** 10.3325/cmj.2011.52.262

**Published:** 2011-06

**Authors:** Danijela Budimir, Ozren Polašek, Ana Marušić, Ivana Kolčić, Tatijana Zemunik, Vesna Boraska, Ana Jerončić, Mladen Boban, Harry Campbell, Igor Rudan

**Affiliations:** 1 University of Split School of Medicine, Split, Croatia; 2 Croatian Centre for Global Health, University of Split School of Medicine, Split, Croatia; 3 Public Health Sciences, University of Edinburgh, Edinburgh, UK

## Abstract

**Aim:**

To systematically assess the existing literature on ethical aspects of human biobanks.

**Method:**

We searched the Web of Science and PubMed databases to find studies addressing ethical problems in biobanks with no limits set (study design, study population, time period, or language of publication). All identified articles published until November 2010 were included. We analyzed the type of published articles, journals publishing them, involvement of countries/institutions, year of publication, and citations received, and qualitatively assessed every article in order to identify ethical issues addressed by the majority of published research on human biobanking.

**Results:**

Hundred and fifty four studies satisfied our review criteria. The studies mainly came from highly developed countries and were all published in the last two decades, with over half of them published in 2009 or 2010. They most commonly discussed the informed consent, privacy and identifiability, return of results to participants, importance of public trust, involvement of children, commercialization, the role of ethics boards, international data exchange, ownership of samples, and benefit sharing.

**Conclusions:**

The focus on ethical aspects is strongly present through the whole biobanking research field. Although there is a consensus on the old and most typical ethical issues, with further development of the field and increasingly complex structure of human biobanks, these issues will likely continue to arise and accumulate, hence requiring constant re-appraisal and continuing discussion.

Biobanks comprise organized collections of human biological samples, usually associated personal health information, which are used together for biomedical research. Research results are generally very important for the society and biobanks have been heavily supported by many governments. Thus, in the recent few years biobanks have undergone rapid proliferation and have become increasingly complex. Their complexity has arisen from an increasingly diverse set of research purposes, and of types and sources of the samples. For instance, biobanks could comprise the collections of human bodily substances of all kinds, such as cells, tissues, blood, or DNA. They range in capacity from small collections of samples to large-scale national repositories. The collected samples could be population-based or disease-specific, originating from diverse profile of individuals, eg, minors or adult persons. Biobanks may contain anonymous human samples or samples linked to the specific personal information. Also, there are various purposes of biobanks, such as diagnostic, therapeutic, or research. Biobanks could be an ownership of public or private subjects, the latter being non-profit or profit based. As a consequence, such a diversity of biobanking is associated with a broad spectrum of ethical and legal issues (1-5).

Ethical issues are commonly present in many aspects of biobanking. The fact that biobanks deal with human samples, invading an individual autonomy or limiting self-control, provokes a number of ethical issues. Who is actually competent to give informed consent and donate a sample? When individuals donate part of their body to a biobank, how is that human sample processed? Who is the owner of the sample? Who should decide how it should be used? Who has the right to know individual results of research? These and many more ethical dilemmas exist in the ethical framework of biobanks. With the recent rapid developments in biobanking, all of these issues are magnified with plenty of further new questions continuously arising. Ethical framework has been the most controversial issue in the domain of biobanking. Thus, it is not surprising that there is a substantial literature focusing on ethical dilemas in biobanking, such as informed consent, privacy, protection, and returning of results to participants (6-9).

Due to these reasons, it is very important that researchers are provided with a current review of the literature on the ethics of biobanking in a systematic way, to document the latest consensus on ethical issues in biobanking and to highlight emerging issues. For that purpose, we reviewed the existing literature on ethical aspects of human biobanks. We aimed to develop a systematic framework for categorizing ethical concerns relevant to human biobanks and to monitor the impact of research into ethical issues. This could help the ethical boards in decision making when dealing with issues within the framework of biobanking. Moreover, we believe that such kind of work could stimulate policymakers and lawmakers to create an adequate legal framework for biobanking, an important, but still largely unregulated issue.

## Methods

### Search strategy

This study was based on a systematic literature search, aiming to identify all relevant studies that fit into the domain of ethical issues in biobanking. We initially searched Web of Science (WOS) database using all combinations of the search terms “ethics OR ethical” AND “biobank*”. In addition, we cross-checked our findings with the PubMed database and searched for additional sources of information. No other limits were set (study design, study population, time period, or language of publication) in order to provide a comprehensive set of results. The search period was from January 1950 to November 2010, and the search was performed in December 2010.

### Selection criteria

We used a four-phase flow diagram from the Preferred Reporting Items for Systematic Reviews and Meta-Analyses Guidelines to improve the quality of inclusion criteria (10). Initially, we included all retrieved articles from both databases, and first eliminated all double entries. All retrieved publications were then manually reviewed. We excluded those that were mentioning both terms (biobanks and ethics), but not necessarily related to each other in the text. Also, we excluded the articles that only marginally mentioned ethics, but it was not the focus of the article.

### Analysis of findings

All included studies were analyzed in two ways. First, we analyzed the type of published work, journals publishing the articles, author’s affiliations (countries and institutions), year of publication, and citations received. Additionally, we qualitatively assessed every article in order to identify ethical issues addressed by the majority of published research on human biobanking. This was done based on categorization of all included publications according to specific independent ethical issues, until saturation of each category was achieved. We reviewed the articles in each category and summarized the main messages, which are described in subheadings in this review. This was done independently by DB and IR, and their results were compared and discussed, until an agreement was reached.

## Results

The results of the search were very similar in both WOS and PubMed databases. The initial search returned 198 articles cited in WOS and 231 articles cited in PubMed in the 1950-2010 period. In spite of no time period limit, all articles were published after 1998, which was expected concerning the time of biobank establishment. There were only a handful of articles in WOS that were not in PubMed, and about 40 articles in PubMed that were not in WOS.

We excluded 22 articles from WOS due to lack of relevance (they mentioned the terms biobanks and ethics, but not related to each other). After a careful reading, additional 28 articles were excluded that did not have ethics as the actual focus. In addition, we examined additional 40 articles found on PubMed and excluded 34 articles. Finally, we retained 154 articles (148 articles from WOS and 6 from PubMed) that addressed ethical issues related to the development and use of human biobanks.

### Scientometric analysis of the included papers

Among the 154 retained articles, most were original research articles (64.2%), followed by review articles (18.2%), editorial material (5.2%), and other less frequently present types (Table 1). They were most commonly found in *Pathobiology*, *European Journal of Human Genetics,* and *Public Health Genomics*, each containing about 5% of the included articles (Table 2). Only 34 articles included investigation of people’s opinion (ie, empirical data), while the rest dealt with theoretical ethical problems and debate of ethical issues.

**Table 1:** Type of published document related to key words “ethics,” “ethical,” and “biobank” during 1999-2010 period

Rank	Document type	Number (%)
1	Original research articles	99 (64.2)
2	Review	28 (18.2)
3	Editorial material	8 (5.2)
4	Proceedings article	7 (4.5)
5	News item	3 (1.9)
6	Book	3 (1.9)
7	Letter	3 (1.9)
8	Meeting abstract	3 (1.9)

**Table 2:** Ten journals most frequently involved in publishing articles related to ethics in developing biobanks during 1999-2010 period

Rank	Journal	Number (%) of articles
1	*Pathobiology*	9 (5.8)
2	*European Journal of Human Genetics*	8 (5.2)
3	*Public Health Genomics*	8 (5.2)
4	*American Journal of Medical Genetics Part A*	5 (3.3)
5	*Journal of Law, Medicine & Ethics*	5 (3.3)
6	*Journal of Medical Ethics*	5 (3.3)
7	*Personalized medicine*	5 (3.3)
8	*Annual Review of Genomics and Human Genetics*	4 (2.6)
9	*Medicine, Health Care and Philosophy*	4 (2.6)
10	*New Genetics and Society*	4 (2.6)

Most of the studies were published in a few highly developed countries (USA, UK, Canada, Sweden, and Denmark). In more than 80% of the articles, at least one author came from these countries (Table 3). Other articles came mostly from southwestern and northwestern Europe, particularly France, Spain, Italy, Germany, Norway, the Netherlands, and Belgium. Apart from a few articles from Australia, articles from other parts of world, such as Eastern Europe, Asia, Africa, or South America, were very rare. The institutions most involved in this discussion were the University of Copenhagen (Denmark), Uppsala University (Sweden), Karolinska Institute (Sweden), and University of British Columbia (Canada), followed by several US-based universities (Table 3). Only 20% of all articles received (or at least acknowledged receipt of) some kind of funding support, mostly from the European Commission (3.3%), National Human Genome Research Institute (3.3%), and European Union (2.6%) (Table 4).

**Table 3:** Contribution of 10 most involved countries and institutions that published titles related to ethics in developing biobanks during 1999-2010 period

Rank	Publication source	Number (%) of articles
	**Country**	
1	USA	53 (34.4)
2	England	25 (16.2)
3	Canada	20 (12.9)
4	Sweden	20 (12.9)
5	France	19 (12.3)
6	Spain	17 (11.0)
7	Germany	15 (9.7)
8	Denmark	14 (9.1)
9	Italy	14 (9.1)
10	Netherlands	12 (7.8)
	**Institution**	
1	University of Copenhagen	12 (7.8)
2	Uppsala University	9 (5.8)
3	Karolinska Institutet	8 (5.2)
4	University of British Columbia	8 (5.2)
5	Facultés De Médecine De Toulouse	7 (4.5)
6	Université de Montréal	7 (4.5)
7	Baylor College of Medicine	6 (3.9)
8	Indiana University	6 (3.9)
9	Katholieke Universiteit Leuven	6 (3.9)
10	Stanford University	6 (3.9)

**Table 4:** Ten most involved institutions that provided support for publishing articles related to ethics in developing biobanks during 1999-2010 period

Rank	Funding Agency	Number (%) of articles
1	European Commission	5 (3.3)
2	National Human Genome Research Institute	5 (3.3)
3	European Union	4 (2.6)
4	FWO Flanders	3 (1.9)
5	Genome BC	3 (1.9)
6	National Institutes of Health	3 (1.9)
7	Canadian Tumor Repository Network CTRNet	2 (1.3)
8	Chair Ethics Office	2 (1.3)
9	Fondos de Investigacion Sanitaria	2 (1.3)
10	Fundación Caja Navarra	2 (1.3)

**Figure 1:**
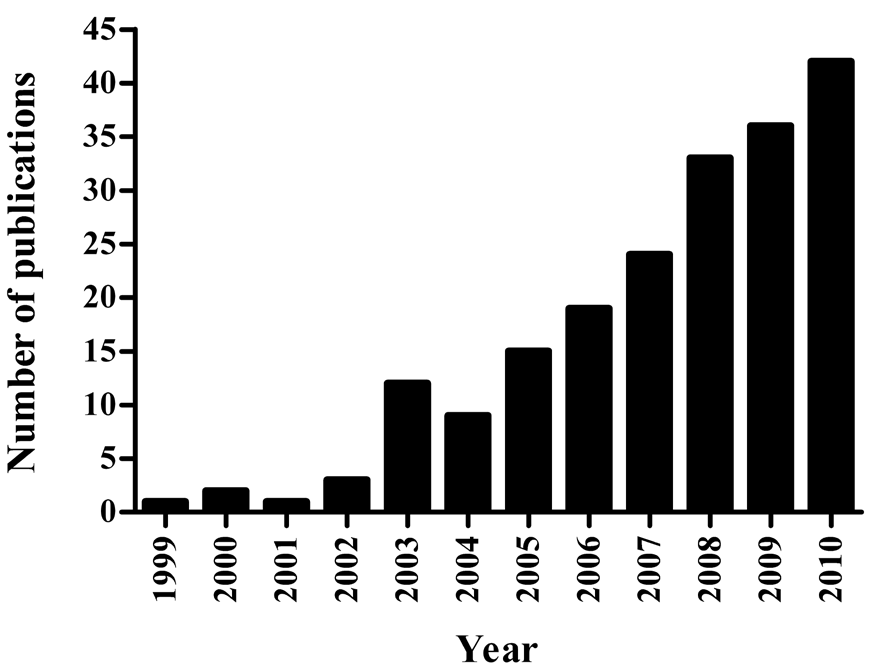
The yearly distribution of published articles related to ethics in biobanks during 1999-2010 period.

**Figure 2:**
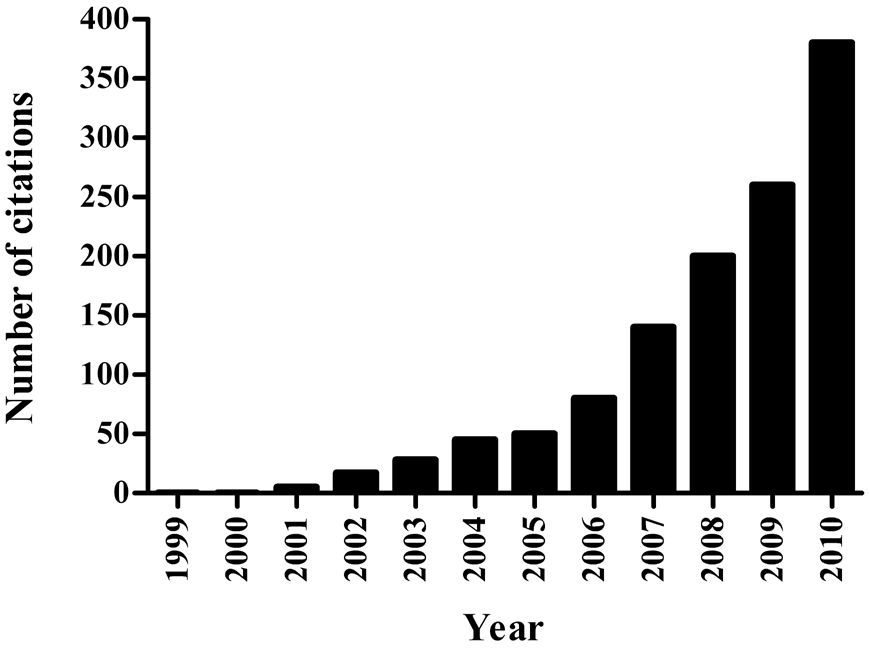
The yearly distribution of citations of publications related to ethics in biobanks during 1999-2010 period.

Although the first biobanks were established nearly two decades ago, more than a half of the retained titles were published in the last two years (Figure 1), illustrating a marked increase in interest. A near-perfect agreement was noticed between the number of articles and the number of citations (Figure 2), which increased greatly in 2009 and 2010. These indicate that ethical aspects of human biobanks are a very fast-evolving field.

We also tried to identify the articles that represented the most intensely cited contributions within this area of research to date. We used WOS to investigate the number of citations received for each study. The analysis of citation was focused on intensity (citations per year), rather than on a total number of received citations, because the latter is largely influenced by the amount of time that elapsed since publication, while the former is a more stable indicator that becomes very informative within the first year from publication. Among 154 articles, only 16 received more than 5 citations per year (ie, about 10%). The topics found in cited articles were most commonly presentation of a few current ethical dilemmas, informed consent, problems in genetic biobanks, and integration of biobanks.

The most intensely cited article was the study of Hansson et al (2006) with 10.40 citations per year (11) (Table 5). This article discussed advantages for accepting a broad consent framework for future research. As we previously found that most articles discussed informed consent and especially its type, it was not surprising that this article was most intensively cited by other authors. The article by Secko et al that also ranked quite high on our list, also discussed informed consent (12). It reported on a public debate over informed consent and offered worthwhile contributions and potential help in the governance of biobanks.

**Table 5:** The most citied articles in the field of ethics and biobanks, which received 5 or more citations per year

Rank	First author	Title	Journal	Year	Cumulative citation	Citations per year
1	Hansson MG	Should donors be allowed to give broad consent to future biobank research?	*Lancet Oncology*	2006	52	10.40
2	Yuille M	Biobanking for Europe	*Briefings in Bioinformatics*	2008	25	8.33
3	Caulfield T	Research ethics recommendations for whole-genome research: Consensus statement	*PloS Biology*	2008	23	7.67
4	Hoeyer K	Informed consent and biobanks: a population-based study of attitudes toward tissue donation for genetic research	*Scandinavian Journal of Public Health*	2004	51	7.29
5	Cambon-Thomsen A	Science and society - The social and ethical issues of post-genomic human biobanks	*Nature Reviews Genetics*	2004	50	7.14
6	Greely HT	The uneasy ethical and legal underpinnings of large-scale genomic biobanks	*Annual Review of Genomics and Human Genetics*	2007	28	7.00
7	Cambon-Thomsen A	Trends in ethical and legal frameworks for the use of human biobanks	*European Respiratory Journal*	2007	27	6.75
8	Gulcher JR	Protection of privacy by third-party encryption in genetic research in Iceland	*European Journal Of Human Genetics*	2000	72	6.55
9	Haga SB	Ethical, legal, and social implications of biobanks for genetics research	*Genetic Dissection of Complex Traits, 2nd Edition*	2008	13	6.50
10	Helgesson G	Ethical framework for previously collected biobank samples	*Nature Biotechnology*	2007	26	6.50
11	Secko DM	Informed consent in biobank research: A deliberative approach to the debate	*Social Science & Medicine*	2009	13	6.50
12	Hansson MG	Ethics and biobanks	*British Journal of Cancer*	2009	12	6.00
13	Kettis-Lindblad A	Genetic research and donation of tissue samples to biobanks. What do potential sample donors in the Swedish general public think?	*European Journal of Public Health*	2006	29	5.80
14	van Veen EB	TuBaFrost 3: Regulatory and ethical issues on the exchange of residual tissue for research across Europe	*European Journal of Cancer*	2006	23	5.75
15	Lee SSJ	Research 2.0: social networking and direct-to-consumer (DTC) genomics	*American Journal of Bioethics*	2009	11	5.50
16	Aalto-Setala K	Obtaining consent for future research with induced pluripotent cells: opportunities and challenges	*PloS Biology*	2009	10	5.00

The article by Haga et al (2008) discussed general ethical dilemmas in human genetic biobanks (13). The article was attractive as it presented the most common ethical issues illustrated by experiences of several national biobanks. Other articles that discussed general ethical problems were also highly cited (7,9), which was expected because they covered numerous current ethical problems.

The article by Heglesson et al (8) discussed the ethics of the use of previously collected samples. Other important and cited articles discussed heterogeneous ethical issues related mostly to genomic biobanking (13-19). Genomic biobanks obviously need to deal with the most controversial ethical issues and provoke extensive discussion. These studies discussed many common ethical issues, all magnified in genomic biobanks, in an empirical or theoretical way and presented current trends or proposed solutions.

It is interesting that among the most frequently cited articles, several were related to proposed mechanisms of integration and harmonization of biobanks (20,21). This implies that both are broadly recognized as desirable, but very difficult to achieve. There is no doubt that harmonization would provide more efficient progress in research, but many ethical and legal problems are yet to be resolved. We believe that this discussion will continue and perhaps increase in future until the most appropriate agreements are achieved.

It is also clear that the research on human induced pluripotent stem cells is increasingly contributing to this debate. The article by Aalto-Setälä et al from 2009 (22) discussed the consent process for the donation of somatic cells to derive pluripotent cells. The article received a relatively large number of citations in a short time period by introducing an emerging ethical problem.

### Content analysis of the included articles

The first aim of the qualitative analysis was to identify separate ethical issues that were addressed by the body of published research. The ethical issue most often discussed was the consent to participate in research. It was mentioned as the only topic in as many as one quarter of all included articles, and as one of several ethical topics in additional 25%. The role of incompetent participants, especially children, was discussed in 15 articles, mostly as the only topic. In general, a larger number of other ethical issues were discussed together. Privacy and identifiability was mentioned in 18 articles, return of results to research participants in 11, and importance of public trust in 10 articles. Only a few studies discussed commercialization, the role of ethics boards, international data exchange, the ownership of samples, and benefit sharing.

We present each of these categories of ethical issues separately. We begin with informed consent since this was the most commonly discussed issue, followed by other ethical issues that emerged to the present date.

### Informed consent

The topic most frequently discussed was consent for participation in research. Consent allows individuals to protect their right to decide whether and how their body parts will be used in research. Accordingly, all authors strongly agreed that participants should not participate and not be exposed to any risk of research without their consent. They also agreed that before obtaining consent, all participants must be well-informed and understand the purpose of research with its expected benefits and risks. Only after that, a voluntary consent can be obtained from each participant. Most of the authors also supported the opportunity of each participant to withdraw their consent (6,23-26), because this ensures additional protection and respect for the participants. However, withdrawal of consent opens further issues: what exactly can be withdrawn, and at which point of study? There is no consensus over this problem at this time (9,25).

Discordance in opinion was also found in relation to the type of consent. Some authors advocated a universal, standardized consent form (27,28), as this would be practical and ensure comparability. Others disagreed and suggested that geographical, social, and religious differences must be respected, together with different research purposes, and that these cannot be addressed in a satisfactory way through an universal and standardized consent form (29,30). Some authors even proposed their own forms of universal standardized consent (27,28,31,32) to facilitate future biobank-based research from the ethics point of view. However, there is still no consensus in the literature about which approach should be adopted.

Nevertheless, most articles focused on the most appropriate content of the informed consent form. Is an *informed* consent, which contains all details about research, the most ethical one? Or can *broad* or even *blanket* consents, which do not contain detailed information, be considered to be satisfactory in some situations? How can the latter be accepted if the purpose of consent is to elucidate all details about research and if they do not provide any specific information? In spite of these concerns, the majority of authors supported the contention that broad consent is the most applicable for the future research, in which the details of research are not known at the time when the consent is obtained. These situations are often present in genetic research. However, authors proposed some conditions that must be respected when using broad consent: research must be of great importance, a maximum protection of privacy must be guaranteed to participants, they must be allowed anytime to withdraw the consent, and every future research should be approved by an ethical review board (ERB) (6,7,11,17,26,33-43). Furthermore, if patients have indicated that they do not wish to participate in any future research, this decision must be respected.

It has been acknowledged that re-contacting the study participants to provide additional or new consent for every future research question or technology can be very impractical, time consuming, expensive, and even confusing (or harassing or worrying) to the participants. Thus, broad consent has an advantage that it does not require re-contact. Only in the case when participants give a broad consent, a re-consent is not required. In all other cases, a re-consent must be sought from participants (17,44).

An important problem that is not often discussed is what should be done in the case of participant’s death? Asking for re-consent is impossible, but should research be continued as it cannot harm the participant anymore? The few authors that addressed this topic offered the view that human samples can be used in every such case, except when there was a previously expressed wish not to use the samples for any future research (6,26,35).

Another concern that was raised was whether it was acceptable to use the samples that did not have consent in cases when it was not realistically possible to obtain it. For example, there are very large collections of human samples collected for diagnostic or clinical purposes that can be useful in research. In most cases, it is impossible to re-contact people to obtain an informed consent, although it would be the most ethical way of respecting their autonomy. But, if re-contact is not possible and these samples are not utilized, the potential for research could be significantly reduced. Therefore, most of the authors agree that the use of such samples in research could be permitted without consent if they are fully anonymized or carry a minimal risk of breaking privacy and thus should not harm the donors; however, every such research must be approved by an ERB (6,8,26,33,34,45-57).

### Privacy and identifiability of the samples

Protection of the identity of research participants is one of the most fundamental ethical issues. The major harm risk in biobanks is associated with breaking privacy (6). The first step in the process of protection, which biobanks broadly accept, is the requirement of informed consent (58). Biobanks, mostly genetic ones, usually store genomic information that is linked to a particular phenotype. That link between two types of information presents a major threat to individual’s privacy (16,59,60). There is a widespread concern that insurance companies and employers could access personal information. They usually have great interest in personal information and biobanks must guarantee adequate protection of personal data. Further, results of research can also harm not only individuals, but the whole groups could feel stigmatized because of their genetic predisposition or other relevant information. Biobanks that perform research on a specific ethnic or other group of people must consider this and be very careful when publishing the results.

These examples warn us that ethical concerns of protection and privacy must be respected in all aspects of biobank framework (61-63). Biobanks have thus proposed several levels of protection for personal data, but none seems to guarantee a complete protection (17). Using anonymous or anonymized samples (no link to other data or a destroyed link) is the best way to protect personal information. But this seriously limits the research utility (16,24,34,64), especially the potential to transform biobanks into longitudinal epidemiological studies. In the case of destruction of the samples, many biobanks could not adequately utilize these data, and genetic biobanks could not utilize them at all. For example, the link between genomic and phenotypic information can be broken. Also, re-contacting specific participants to provide new informed consent is unachievable. There is no possibility of returning the results. Withdrawal of consent also becomes impossible to carry out in practice. So, many authors refuse permanent anonymization and support coding of information as the most appropriate way of ensuring privacy. There is an agreement that simple coding, double-coding, or even triple-coding (one to three codes are needed to provide a link between sample and data) are acceptable in standard research practice, and at the same time are safe enough to ensure a satisfactory level of privacy (7,17,64-68).

This fundamental concern about privacy is usually also the main concern of the participants when they are deciding whether to donate their samples to biobanks. One study showed that up to 90% of people were concerned about their privacy (69). Thus, consequences of breaking privacy could substantially affect public’s willingness to participate and substantially delay the research. Therefore, biobanks must always guarantee a maximal level of protection of participants (6,70).

### Returning the results to the examinees

A general ethical standard requires that each individual has “the right to know and the right not to know” the results of research. A result can be of statistical, clinical, and research interest, but also can be incidental. So, which results of research should be returned to participants of the study? When should they be returned, and under what circumstances? How should they be returned and communicated and by whom (with what level of training)?

The issue of return of results has sparked a broad debate about ethical implications of biobanking. Although very few articles confirm that participants actually have a substantial level of interest in getting their personal research results back (71,72), it appears that most of the authors do not advocate returning the individual results (6,7,9,17,26,34,59,73,74). They warn that returning the information can be misinterpreted and cause anxiety among the participants, especially if information is not of any clinical relevance. Misinterpretation is referred to clinically irrelevant result, or results that are not yet validated, understood properly, or informative. These results can cause psychological, social, or economic harm to participants. But, if a result is clinically important, is it ethical not to return it? Furthermore, how are clinically important results best defined?

Most of the authors agree that the only exception to the general rule of not returning the results can be a result of very high clinical importance. Such a result should be returned and communicated properly and professionally to each participant. This could be in the form of either a research finding or an incidental finding. There needs to be an effective mechanism of prevention available, or a proper treatment, which could substantially improve or even save someone’s life. However, the main purpose of biobank-related research is not genetic counseling of donors, but rather increased knowledge leading to a longer term collective improvement of health. Researchers involved in biobanks have an obligation to publish all relevant scientific information that could help the society as a whole.

In practice, the governing policy of most biobanks is not to return any individual results to their participants. However, a number of authors agree that this is not always ethical (17,75). Biobanks should consider returning the findings of high clinical relevance to participants. However, it is also acknowledged that the policy of not returning the results often provides a kind of protection against incorrect results (perhaps due to less stringent quality control measures than are found in an accredited clinical laboratory) and against results causing harm to the participant.

### Ensuring and sustaining public trust

The most important prerequisite for successful biobank-related research is ensuring the public trust. This can be achieved through continuous education of people and protection of privacy. Depletion of public trust can have damaging consequences in biobanking, but the main victims are the people who expect improvements from medicine and health systems (6).

All the reviewed articles that investigated public opinion on biobanks confirmed that people were generally enthusiastic and supportive of biobanks and were genuinely willing to participate (15,19,69,76-82). Some of them did not really understand the actual purpose of biobanking, but they wanted to participate nevertheless (83-86). Several articles also confirmed that people who were more educated and informed about biobanking were also more willing to participate (12,87). Many deliberative events in Canada are thus being held regularly to ensure public trust and facilitate their engagement (12,88-90). During such events, people are encouraged to learn, debate, and discuss the aims and goals of biobanks. All articles confirmed that these events were useful in stimulating the citizens and providing greater public engagement in research, but also helped in structuring the governance of biobanks (12,88-90).

It seems that at the present time biobanks have admirable levels of support from the public. However, they must continue to maintain and improve levels of public trust. Providing consent and protection of privacy are obligatory elements of that process (91,92). As a few articles confirmed, ERB are especially important as subjects of public trust (6,15,19,93) and they often play an exceptionally important role in the biobank success.

### Children and incompetent adults as study participants

Involving children in biobanks raises ethical dilemmas that are not entirely analogous to adults’ involvement. Children (or more precisely – minors) have a limited capacity to understand the ethical and other issues surrounding the biobanks and thus represent the most vulnerable population. The majority of biobanks do not involve children because of special ethical problems and concerns that are not easily addressable, and also because the increased sensitivity of the public and the media toward this segment of the population sometimes makes it an unnecessary risk that many biobanks are not willing to take. However, this could lead medical research on children to lag behind the research on adults. From the ethical point of view, in that way children will eventually suffer relatively more than adults. So, nearly all authors support the idea of involving children in biobanks, but they also agree that the risk for them should be actively minimized (94-101). Because children require extra protection, some authors even suggested that data sharing on children should be banned until children reach adulthood and give specific consent to share their samples from population databanks (102). However, other authors strongly opposed this suggestion, arguing that such proposal would seriously delay research on children – possibly leaving the whole generation behind (103-105).

Furthermore, although parents will be expected to be very interested in having the results of research findings on their children returned to them (96), most of the authors agree that this would not be an ethically acceptable policy (96,106). Parents do have a right to decide whether they want to involve their children in a biobank and they also have a right to give informed consent instead of the children. However, the children must decide if they want to know about their own results when they reach adulthood. Another cause for concern is that, if parents knew some genetic information of clinical relevance later in life, they could demand treatments for children as if they already had the condition, in order to protect them. But, what should be done with incidental findings that could potentially save a child’s life? Not many authors discussed this issue, but one article (106) suggested that it would be ethical to return this information to parents.

Besides obtaining parental informed consent, it is a general opinion that researchers must also ask for consent from children whenever it is possible (95). In some instances, research can be conducted without parental or child’s consent. These situations are rare, but must ensure minimal or no risk for children. Researchers must always respect the child’s fear and/or disagreement to participate. An ethical board must be consulted in every research study carried out on children in order to protect the interests of the child (95,97,107).

Although it is only rarely mentioned in the literature, similar ethical principles also apply to incompetent participants – for example, those suffering from psychiatric diseases. They must always be closely protected and exposed to an absolutely minimal risk. A guardian must sign informed consent and an ethical board must be consulted in all cases to protect the interests of incompetent adults (26,108).

### Commercialization

Although biobanks have a primary focus on research and improving medical knowledge, this will not necessarily prevent private companies from trying to use biobank data for their own interest. In general, commercialization raises several ethical issues, such as preventing exploitation, ensuring fairness to study participants, and balancing costs and benefits (59). Some articles showed that commercialization, in general, tended to decrease public trust in biobanks (26,34,69), although it did not completely diminish it.

A typical example of commercialization in biobanking is pharmacogenomics research supported by pharmaceutical companies (26,109,110). They support research that could eventually improve treatment, but they also hope that the results of such research could prove very profitable in the future. Also, gene patents are potentially very profitable, so many companies are willing to support such investigation to achieve future profits (9,111). But, is it ethical to create such financial benefits from free donations and who has the right to a share in these profits? How should costs and benefits be balanced and how should intellectual property be shared between companies, researchers, and participants?

These dilemmas have not been resolved in the literature. In fact, given how quickly some major players in the private sector are moving toward commercialization, there is insufficient discussion in the present literature about these very real and pressing (and not merely hypothetical) issues. We believe that people generally understand that the partnership of research and commercial interest could also be very productive and should not be seen as a threat to their interests. Ongoing discussion on these issues, based on some concrete examples from real life – both positive and negative – is certainly needed.

**Role of Ethics Review Boards**

The role of ERBs will not necessarily be the same in all institutions (6,112,113). ERBs work under ethical and legal frameworks and national legislation, which provide protection of participants and ensure appropriate use of their samples (114-117). As mentioned previously, the role of ERBs is crucial for ensuring public trust. ERBs are safeguards of participants’ interest and thus have a very important role in the process. Also, the whole development of biobanks and their integration depends on ERBs. Despite that, one study showed that members of ethical boards are not necessarily comfortable or satisfied with their present role in biobanks. They want to be more engaged in the whole process of biobanking (118). But, this decision lies with institutions. As ERBs play such a critical role, we believe that there should be more literature that could provide guidance and help improve the quality of their contribution in the process of reviewing biobanking activities.

### Data exchange

Many authors agree that international collaboration is extremely useful and that it should be encouraged, as long as it exposes donors to minimal risk. Data exchange between research groups who work on different biobanks can facilitate research, but it also requires a removal of a major barrier – different design of biobanks in different settings. Thus, many authors see a need for greater international harmonization of the principles of biobanking and its design, and a major role for ERBs in the regulation of that process (6,7,26,119,120). Several institutions have already established national and international cooperation rules and norms (5,26,121-127). Some of these rules are even published in detail (20,21). The main ethical issue of integration and harmonization of the data sets that biobanks contain is the protection of participants’ privacy. This is why ERBs must have an important role in overseeing this harmonization (6).

There is also a matter of digital data in biobanks, especially sharing and storing the data in public repositories. Many journals have over the past few years established the need for making the research data publicly available, raising mixed feelings on the issues related to confidentiality (128). An overview of the journal policies indicates that some journals (such as notably *Genetics*) actually require all the materials to be made publicly available before publication of the research results. This requirement can make strong impact on the research output, since disclosure of this type of information for some types of biobanks (ie, isolated populations) can present a potential and true risk for confidentiality breach. If we assume that publication of research results requires public disclosure of all material, including genetics and even pedigree information, one can envision that the use of a relatively small number of genetic markers and cross-comparison with the pedigree can lead to serious confidentiality breach and possibility to misuse such information. The very existence of such mechanisms is questionable; since another study showed that even when less strict mechanisms were in place, researchers are reluctant to share the raw data in order to protect participants’ confidentiality (129).

### Benefit sharing

Benefit sharing is a very sensitive issue and it is rarely discussed. Benefit from the research results, especially the financial benefit, could be shared among participants, communities that take part in research, researchers, and their institutions. There is a substantial interest from the industry in this research, so that generation of intellectual property and benefits over time is not unlikely. In terms of benefit sharing, biobanks must strike a balance between many competing interest from various stakeholders in the process (9).

There is a high level of agreement between the authors that donors of biological materials should not be paid. Donors will eventually profit from the results that will improve future diagnosis and treatment of the diseases in the population (130). It is suggested that all negative reactions that may raise among the donors could be prevented by professional and responsible information sharing, education of the donors about the research process, and complete transparency over the outcomes of the research in terms of both new knowledge that was generated and the income and its distribution (59).

### Ownership of the biological samples and data

Another infrequently discussed topic in the literature is ownership of samples. It presents ethical and legal issue in biobanking. What happens when a participant donates a part of body to a biobank? Could biobanks become owners of the sample or does it remain in the ownership of the participants? One author has recently explored this issue in great depth and concluded that the legal position on ownership remained unsettled (26). Other authors take the position that complete anonymization would practically make biological materials ownerless, but that in all other instances the donors maintain ownership and should be able to withdraw both their consent and their biological material donated to the biobank (131,132).

Most biobanks have agreed to be custodians or trustees, instead of owners, of samples (130,133-135). One author suggested that samples should be the shared property of donors, researchers, and institutions (64). But, this issue clearly remains insufficiently discussed and it is unresolved.

### A comparison of positions of different stakeholders (eg, ethical boards, research participants, researchers, and institutions)

In the biobank development process, it is extremely important to consider the different opinions of all stakeholders involved in the research process. Many articles have investigated opinions of the ERB, researchers, medical staff, and potential participants in order to help build ethical and legal frameworks for biobanks.

The greatest number of studies was conducted to investigate participants’ attitudes. Generally, participants have positive attitudes toward biobanks and many people are interested in participating (15,19,69,76-79,136,137). They believe strongly in the better future that biobank-related biomedical research could offer to them and their offspring. In addition, several studies investigated opinions of pregnant women about storing their children’s samples in pediatric biobanks. Most of them shared positive attitudes toward pediatric biobanks (80-82). In spite of that, participants’ concerns were always present, such as adequate protection of privacy (69). One of the frequently reported problems was that they did not understand the aims of research fully (83-86), but nonetheless they still agreed to participate. One study discovered that the domains of the information sheet/consent form that were best understood were the nature and benefits from the study and voluntary nature of participation (138). Less understanding was reported for risks of involvement, confidentiality, and experimental nature of research. Expectations of participants were generally similar to this. Most of them wanted control over their access to information, return of the results to them, and involvement of ERBs (15,71,72,93). One study (139) focused on investigating the opinion of the selected sub-sample of the people who refused to donate their samples for genetic research. The main concern was their general mistrust over sharing of their DNA information.

Comparisons of attitudes toward biobanks between the general public and technical experts showed that they were quite different. For example, one article clearly showed that ERB members, researchers, and participants had a rather different opinion about which information should be included in the consent form (140). Generally, experts were more positive about biobanking because they had greater knowledge of the issue than non-experts and they all shared very positive attitudes (87,141). However, even the experts did not always share their enthusiasm toward all aspects of biobanking (134). Similar findings were noted by ERB members: although they would often disagree on many issues relevant to biobanking, a broad general agreement was nearly always present among them (112,118,142).

Only one article interviewed leaders of ethno-cultural communities, who are a relatively frequent category of sponsors and beneficiaries of biobanks (143). The authors concluded that involving leaders in development of biobanks can be very useful. Also, educating them about aims and scope of biobanking is essential for the success and sustainability of research projects. The authors concluded that an improved approach to education of community leaders would substantially improve the whole process of biobanking.

### Legislative framework for biobanks and other emerging issues

In the most developed countries, governments are beginning to pass the formal legislation that governs the principles of development and utilization of biobanks with human samples. One of the most advanced in this regard is Iceland, where the DeCode Genomics Company has been utilizing the genetic heritage, phenotypes, and deep genealogies of most living Icelanders. The Icelandic government has passed “The Biobank Act” to regulate this unusual public-private partnership (144). This gave the researchers from Iceland substantial returns in terms of research competitiveness, but the financial benefits have not been reciprocal for their industry partner. Other countries where a substantive legislative framework is being developed include France, Estonia, Spain, Scandinavian countries, the United States, and the United Kingdom (145-150).

One of the interesting strategic debates relevant to the development of national biobanks was whether it was better to invest in the existing large collections of biologic materials from longitudinal epidemiological cohorts and enrich it with additional measurements to harmonize several existing data sets or to build them brand new and from the start. While the debate over this issue in the USA has possibly delayed research progress and handed the competitive edge to the Europeans, even if only temporarily, the Dutch government supported the existing biobanks (151), while the UK government invested in the entirely new “UK Biobank” with at least 500 000 participants (152,153). In France, an interesting idea was proposed that a massive biobank could be developed very quickly and cost-effectively by recruiting people who volunteer to give blood in transfusion centers, as they would also be likely to take part in biobanking research (154). Other ideas included setting up biobanks as charitable trusts (155).

## Discussion

In this systematic review, we assessed the quantity of research on ethical aspects of biobanking and systematically addressed the most important ethical dilemmas. We attempted to remain impartial on all ethical issues and present how different issues are understood and viewed by different authors. We described how ethical guidelines were viewed among different authors and institutions, but the main ethical principles of respect, benefice, justice, and minimizing harm remained a constant motive. Generally, we observed that there was a consensus on these well-recognized ethical issues, but rapid biobank development continues to provide new issues that are often difficult to follow and discuss. Many issues have created dilemmas that are yet to be resolved, and the consensus still needs to be reached.

Different ethical issues can sometimes arise from different types of biobanks – or sometimes the same issues, but to different extent. It is clear, for example, that the return of an epidemiological result to the participant will not provoke the same level of debate as the return of his/her genetic information. Similarly, collecting cancer samples is not associated with the same level of concern as, for example, collecting of the samples to be used in creation of pluripotent cells that will be transplanted into humans. The biobanks that deal with the latter nature of work are probably the most controversial issue in this domain. In addition to this, prior to the development of genetic biobanks, ethical issues in biobanking were rarely discussed in the scientific literature. Collections of human tissues have a very long history, but they did not raise nearly as many ethical concerns throughout decades of research as biobanks did in the past few years. Post-genomic era exposed and augmented many existing ethical problems and also brought to surface some new ones. With enormous advances in genetic research, ethical issues are changing over time together with development of science and technology. We believe that this trend will continue in the future, but the directions that this research will eventually assume, and the ethical issues that may still arise, are very difficult to anticipate. Therefore, there is a need for enactment of legislation regarding the collection and use of human biological materials and associated data.

Ethical issues in biobanks have emerged as important issues in publishing research from such sources. International Committee of Medical Journal Editors (*http://www.icmje.org*) does not address ethical issues related to publishing results from biobanks. There is no guideline for reporting results from biobank research, as judged by the information on Enhancing the Quality and Transparency of Health Research (EQUATOR) network. These organizations could use this systematic review to address the most important issues related to biobanks and data publishing. However, there is some relevant information at Minimum Information for Biological and Biomedical Investigations (*http://www.mibbi.org/index.php/MIBBI_portal*). There is another similar guideline concerning the quality of reporting in genetic association studies results. A group of experts proposed guiding principles for reporting genetic results as an extension of the previous STROBE Statement and called it the STrengthening the REporting of Genetic Association Studies (STREGA) Statement (156). The STREGA recommendations are available at *http://www.strega-statement.org/* and all journals are endorsed to use them. Some official guidelines or recommendations concerning research on human participants are developed, such as Convention on Human Right and Biomedicine, created by the Council of Europe in 1997 (*http://conventions.coe.int/Treaty/Commun/QueVoulezVous.asp?NT=164&CM=8&DF=23/04/2011&CL=ENG*) and the International Ethical Guidelines for Biomedical Research Involving Human Subjects, created by the Council for International Organizations of Medical Sciences in 2002 (*http://www.cioms.ch/publications/guidelines/guidelines_nov_2002_blurb.htm*). Both relate to the main ethical principles on humans in medical research. In addition to this, the OECD Council in 2009 adopted a Recommendation on Human Biobanks and Genetic Research Databases (HBGRD), the guidelines for the management, governance, access, and use of HBGRDs in a manner respectful of participants (*http://www.oecd.org/sti/ biotechnology/hbgrd*). Since additional official guidelines or recommendations specifically addressing biobanks should be developed, one of the conclusions of this study is to raise researchers’ awareness and consider creation of the biobank-related publishing guidelines.

Implications of the ethics discussion of biobanks are interesting not just for the sake of biobanks themselves, but also for the very foundation of the future medicine. The future will inevitably bring personalized medicine, which will share a number of similarities with the contemporary biobanks – need to protect sensitive information, levels of accessibility, the need to prevent data misuse, and the possibility to predict individual health-related outcomes based on the genomic information.

After a systematic analysis of all articles published in the last two decades that were concerned with ethical implications of biobanking, we conclude that the focus on ethical aspects was strongly prominent throughout the whole biobanking research field development, albeit published by research groups in a very limited set of (highly developed) countries and their leading academic institutions. Ethical issues in conjunction with legal frameworks are major steps in biobanking development, but many unresolved problems remain. With further development of this field and the increasingly complex structure of human biobanks, these issues will likely continue to arise and accumulate, hence requiring constant re-appraisal and discussion on ethical issues.
